# Nivolumab induced myxedema crisis

**DOI:** 10.1186/s40425-017-0213-x

**Published:** 2017-02-21

**Authors:** Uqba Khan, Humaira Rizvi, Dahlia Sano, Jane Chiu, Tarik Hadid

**Affiliations:** Department of Hematology/Oncology, St. John Hospital and Medical Center, 19229 Mack Ave Suite 23 Grosse Pointe Woods, Detroit, 48236 Michigan USA

**Keywords:** Nivolumab, Immunotherapy, Myxedema crisis, Hyperthyroidism

## Abstract

**Background:**

Nivolumab is an anti-programmed cell death (anti-PD-1) monoclonal antibody that is approved by Food and Drug Administration for treatment of metastatic non-small cell lung cancer, metastatic melanoma, relapsed Hodgkin lymphoma and advanced renal cell cancer. We report a rare case of myxedema crisis induced by nivolumab in a patient with metastatic squamous cell carcinoma of lung.

**Case presentation:**

Fifty three-year old woman with metastatic squamous cell carcinoma currently on treatment with nivolumab presented with diffuse facial and tongue swelling, slurred speech, depressed mentation, fatigue and weakness. Initial evaluation revealed severe hypothyroidism with thyroid stimulating hormone of 237 micro Unit/mL (Normal Reference range: 0.27–4.20 micro unit/mL) and undetectable free T4. Patient was diagnosed with nivolumab induced myxedema crisis. She was treated successfully with levothyroxine with complete resolution of her symptoms. Nivolumab was safely restarted once the symptoms of myxedema resolved.

**Conclusion:**

Nivolumab can cause immune-mediated endocrinopathies including thyroiditis, hypophysitis, adrenal insufficiency and type 1 diabetes mellitus. High index of suspicion and periodic measurement of thyroid function tests are recommended in patients receiving nivolumab therapy. Our case also suggests that once the myxedema crisis is treated and symptoms are resolved, nivolumab can be safely re-challenged.

## Background

Cancer treatment has rapidly advanced over the recent years with exodus of newly approved therapies for various malignancies. Immunomodulatory agents such as nivolumab have a unique mechanism of action, dramatically different from that of traditional chemotherapy. As a result, toxic effects are also unique and predominantly immune mediated. Nivolumab is one example of immunotherapy drugs, which is approved by Food and Drug Administration for treatment of metastatic non-small cell lung cancer, metastatic melanoma, relapsed Hodgkin’s lymphoma, metastatic head and neck cancer and advanced renal cell cancer. Although a number of immune mediated adverse effects of nivolumab are reported but myxedema is a very rare adverse effect [1]. Herein, we present a rare case of nivolumab-mediated myxedema in a patient with non-small cell lung cancer (NSCLC).

## Case presentation

A 53-year-old woman was diagnosed with metastatic squamous cell carcinoma of lung with involvement of the liver and mediastinal lymph nodes. She has a history of hypertension, chronic kidney disease due to prior cisplatin use and uterine fibroids but no history of thyroid disease or any other autoimmune disorders. She was receiving second-line therapy using nivolumab, to which she achieved excellent response and complete resolution of liver disease and significant response in the lung and mediastinal lesions. Programmed death-ligands 1 (PD-L1) were expressed on 81–90% of malignant cells. She was doing well until she presented to the emergency department with slurred speech, progressive diffuse facial, periorbital swelling and tongue swelling over the last few weeks. She also complained of generalized weakness, fatigue, forgetfulness, constipation, dyspnea, slow voice, cold intolerance and dry skin. Physical examination revealed slightly overweight woman with cushinoid appearance, depressed mentation, poor memory and delayed relaxation phase of deep tendon reflexes. Facial examination revealed diffuse facial, periorbital and neck swelling. She has no thyromegaly or lymphadenopathy. The rest of the examination was unremarkable.

Laboratory findings showed hemoglobin of 87 gm/L, mean corpuscular volume of 94 fL, white blood cell count of 4.6 × 10^9^/L, platelets of 238 × 10^9^/L, creatinine of 209.5 μmol/L, sodium of 136 mmol/L, potassium of 4.4 mmol/L and calcium of 2.5 mmol/L. Thyroid function tests revealed thyroid-stimulating hormone of 237 micro Unit/mL and free T4 ˂1 pmol/L. Cortisol level was 303 nmol/L. Creatine kinase was elevated at 23.80 μkat/L suggestive of myopathy due to severe hypothyroidism. Computed tomography of the brain revealed no evidence of intracranial metastasis.

These clinical and laboratory findings were consistent with myxedema crisis. Therefore, she was hospitalized and immediately initiated on 100 μg of intravenous (IV) levothyroxine for 3 days. This was followed by 137 μg of oral levothyroxine daily. In addition, she was started on IV hydrocortisone which was subsequently discontinued once adrenal insufficiency was ruled out. She expressed dramatic recovery of her symptoms and laboratory studies. Nivolumab was suspected to be the culprit of myxedema in this patient and was held until symptoms improved. She was subsequently restarted on nivolumab without recurrent symptoms. On continued follow up, she was given another 10 cycles of nivolumab over 5 months period without any signs or symptoms of myxedema.

## Discussion and conclusions

Cancer treatment has revolutionized in recent years. Development of new therapies especially immunotherapy has greatly impacted the outcome of malignant disorders. However, the toxic effects associated with these agents are dramatically different from those encountered with chemotherapy.

There are various immune checkpoints pathways exist which limit the immune response of the body to reduce autoimmunity. One such pathway is programmed cell death pathway (PD-1), which acts as a checkpoint to reduce T-cell mediated immune responses [2, 3]. PDL-1, which are present on tumor, attach with the PD1 receptors on T-lymphocyte and induce signals causing reversible inhibition of T cell activation and proliferation [2]. By blocking this interaction of PDL-1 with PD-1, immune response can be mounted against malignant cells.

Nivolumab is an anti-programmed cell death (anti-PD-1) monoclonal antibody. The Food and Drug Administration (FDA) approved this agent for treatment of metastatic non-small cell carcinoma, metastatic melanoma, relapsed Hodgkin’s lymphoma, metastatic head and neck cancer and advanced renal cell carcinoma [4]. Nivolumab is a well-tolerated agent with the most common side effects being fatigue, musculoskeletal pain, decreased appetite, cough, and constipation. Nivolumab is also associated with several immune-mediated toxic effects such as colitis, pneumonitis, hepatitis, nephritis, skin-rash and endocrine glands dysfunction. Immune-mediated endocrinopathies include thyroiditis, hypophysitis, adrenal insufficiency and type 1 diabetes mellitus.

Thyroid dysfunction is seen infrequently in patients receiving nivolumab. Both hyperthyroidism and hypothyroidism can occur in patients receiving nivolumab. In clinical trials, grade 1 and 2 hyperthyroidism occurred in 1.4–4.4% of patients receiving nivolumab [5–7]. Grade 3 hyperthyroidism is reported in one patient with metastatic melanoma receiving nivolumab [8–10]. The median time to its occurrence was about 2–3 months [11]. Grade 1 and 2 hypothyroidism is estimated to occur in 7–9% of patients receiving nivolumab [5–7]. Grade 3 hypothyroidism was reported in 3 patients, one with metastatic melanoma and two with renal cell carcinoma [11, 12]. The median time to development of hypothyroidism was 2.9 months. It is recommended to check TSH and free T4 before each treatment.

Management of nivolumab-induced hypothyroidism depends on severity of the disease. If hypothyroidism is mild, simple replacement of levothyroxine is sufficient. If hypothyroidism is moderate or severe as in our case, management often requires multidisciplinary approach to avoid life-threatening cardiac complications associated with levothyroxine replacement. It is recommended to hold nivolumab in patients with severe hypothyroidism but it can be reinstituted once the patient is stabilized, as in our patient.

Checkpoint inhibitors especially nivolumab have revolutionized the treatment of various hematological and solid malignancies. It is not only important for oncologists but as well as for general practitioners to be familiarize with unusual adverse effects of these drugs. Immune mediated thyroid dysfunction remains an important adverse effect of checkpoint inhibitors. It has become our routine practice to check baseline thyroid function tests and monitor them periodically during the nivolumab treatment. It is recommended to check TSH and free T4 before every cycle of nivolumab.

## Abbreviations

FDA: Food and drug administration
gm/L: Gram/liter
ml: Millilitler
mmol/L: Millimole/liter
NSCLC: Non-small cell lung cancer
PD-1: Programmed cell death 1
PD-L1: Programmed death-ligands 1
pmol/L: Picomole/liter
μg: Microgram
μkat/L: Micro katal/liter
μmol/L: Micromole/liter
